# The bridge-like lipid transfer protein (BLTP) gene group: introducing new nomenclature based on structural homology indicating shared function

**DOI:** 10.1186/s40246-022-00439-3

**Published:** 2022-12-02

**Authors:** Bryony Braschi, Elspeth A. Bruford, Amy T. Cavanagh, Sarah D. Neuman, Arash Bashirullah

**Affiliations:** 1 HUGO Gene Nomenclature Committee, European Molecular Biology Laboratory, European Bioinformatics Institute, Hinxton, Cambridgeshire, CB10 1SD UK; 2 Department of Haematology, School of Clinical Medicine, University of Cambridge, Cambridge, Cambridgeshire, CB2 0AW UK; 3 Division of Pharmaceutical Sciences, University of Wisconsin-Madison, Madison, WI 53705-2222 USA

## Abstract

The HUGO Gene Nomenclature Committee assigns unique symbols and names to human genes. The use of approved nomenclature enables effective communication between researchers, and there are multiple examples of how the usage of unapproved alias symbols can lead to confusion. We discuss here a recent nomenclature update (May 2022) for a set of genes that encode proteins with a shared repeating β-groove domain. Some of the proteins encoded by genes in this group have already been shown to function as lipid transporters. By working with researchers in the field, we have been able to introduce a new root symbol (BLTP, which stands for “bridge-like lipid transfer protein”) for this domain-based gene group. This new nomenclature not only reflects the shared domain in these proteins, but also takes into consideration the mounting evidence of a shared lipid transport function.

## Introduction

For over 40 years the HUGO (Human Genome Organisation) Gene Nomenclature Committee (HGNC) has been assigning standardised nomenclature to human genes [1, 2]. As genomics is being increasingly used in a clinical setting, the need to utilise a common language when referencing genes has become ever more important [3]. Stabilising gene symbols is now a key priority for the HGNC, but there are still some cases where an update to a more functionally informative nomenclature, when supported by the community working on the gene or genes in question, can be justified. In particular, we are still working to update uninformative “placeholder” symbols such as C#orfs, KIAAs and FAMs as long as they have not become entrenched in the literature [1]. As research reveals functional information about the proteins encoded by previously uncharacterised genes, this enables us to assign a new and informative nomenclature, which can then be stabilised.

## The initial nomenclature update consultation: KIAA1109

The HGNC initiated a consultation in February 2022 about a potential nomenclature update for the gene previously approved as *KIAA1109* (HGNC:26953). When we propose an update for an uninformative placeholder symbol, we write to authors who have published on the gene in question, wherever possible [1]. A previous consultation in 2020 had failed to reach a consensus agreement on new nomenclature, as some researchers felt that at that point there was not yet enough direct evidence to name *KIAA1109* based on the possible role of its protein product in endocytosis [4, 5].

The first ortholog of *KIAA1109* identified was Csf1p in budding yeast, named for the cold-sensitive fermentation (CSF) phenotype arising from gene disruption [6]; a molecular explanation for this phenotype was lacking for over 20 years [7]. A metazoan ortholog of *KIAA1109* was first characterised in *Drosophila*, and its encoded protein was referred to as “Tweek”. This name originated from the fly neurological phenotype that was associated with seizures and was reminiscent to researchers of a jittery cartoon character. The fly protein Tweek was found to be involved in endocytosis and was required for synaptic vesicle recycling, affecting the availability of phosphoinositide lipids at synapses [5]. Tweek was reported to play a role in regulating a WASp (Wiskott-Aldrich Syndrome Protein)/PI(4,5)P_2_-dependent pathway, and a separate signalling pathway involving the fly proteins encoded by *nwk* (nervous wreck) and *wit* (wishful thinking), both of which are regulators of synaptic growth [8]. These genes are conserved in humans (with *FCHSD2* (FCH and double SH3 domains 2) and *BMPR2* (bone morphogenetic protein receptor type 2) being the orthologs of *nwk* and *wit*, respectively), and FCHSD2 has been shown to play a role in endocytosis [9], suggesting that the pathway in which Tweek functions may be conserved.

Variants in the human *KIAA1109* gene have been associated with the congenital neurological malformation disorder Alkuraya-Kucinskas syndrome (MIM number 617822) [8]. To characterise cellular phenotypes upon mutation of *KIAA1109*, primary dermal fibroblasts from a patient and a control subject were taken, and immunofluorescence techniques and endocytosis assays were used to examine the endosomal, cytoskeletal, and ciliary cellular phenotypes associated with this condition. The findings of Kane et al. suggested that this syndrome is associated with pleiotropic defects in the endocytic pathway, the actin cytoskeleton and primary cilia [10].

Gene nomenclature in fly can be very different to that of vertebrates; it is often based on phenotypes and may sometimes aim to be humorous or to reference pop culture or literature, e.g., flies with a variant of the *tinman* gene have cardiac deformities [11, 12], referencing the tinman from the Wizard of Oz who lacks a heart. Many such names are unsuitable for transfer across to their human orthologs, as they could be potentially perceived as pejorative if associated with a phenotype. Understandably, “TWEEK” is among this group, and after discussion with researchers about the nomenclature of *KIAA1109*, we felt that they appreciated why we could not approve “TWEEK” as the symbol for this gene in human and across vertebrates, although we were able to add “Tweek” to the gene record as a published alias.

## A new root symbol proposal: BLTP

**Fig. 1:**
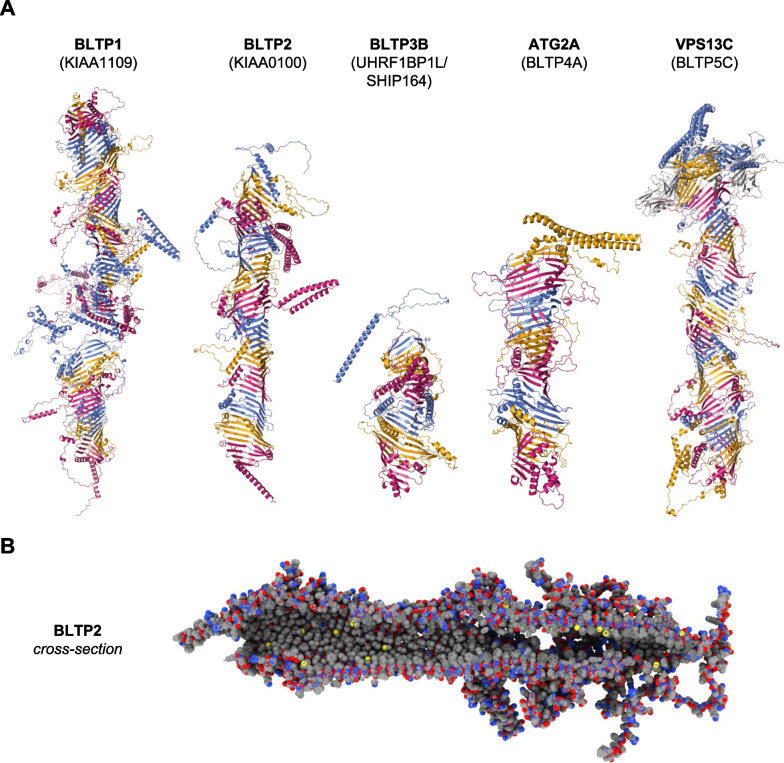
Structures of the human bridge-like lipid transfer protein (BLTP) superfamily members. **A** Ribbon models of the predicted structure of representative members of the human bridge-like lipid transfer protein (BLTP) superfamily. Repeating β-groove (RBG) domains are alternately labelled in pink, orange, and blue; the VAB domain of VPS13C [17–19] is shown in grey, and the ATG_C and PH domains [13] are shown in teal. Note that the positioning of these domains in VPS13C relative to the hydrophobic groove has not been experimentally determined. Large unstructured loops have been omitted from BLTP1, BLTP3B and ATG2A for visual clarity. HGNC approved symbols (in bold) are used for the encoded proteins; previous symbols/aliases are in parentheses. **B** Sphere model showing a cross-section of BLTP2 with carbon atoms (grey), oxygen (red), nitrogen (blue), and sulphur (yellow). Note the presence of an inner hydrophobic groove ideal for transport of lipids. The predicted structures of BLTP2, BLTP3B and ATG2A were downloaded from the AlphaFold database [12]. The VPS13C structure was generated using trRosetta [14] following the protocol described in [17]. The BLTP1 structure was generated by using trRosetta [14] to fold seven ~ 1500 amino acid overlapping fragments of the protein; these fragments were then assembled using the *matchmaker* command with Needleman-Wunsch global alignment in ChimeraX v.1.4 [20]. All ribbon models were coloured and rendered using PyMOL v.2.4.0; sphere model rendered using ChimeraX v.1.4 [20]

The release of protein structures in the AlphaFold database [13] and modelling with trRosetta [14] highlighted key structural features of KIAA1109 that enhanced understanding of the putative function of this protein [15, 16]. Additionally, analysis of predicted structures led to the identification and classification of the superfamily of proteins of which KIAA1109 is a member: the bridge-like lipid transfer proteins (BLTPs) (Fig. 1).

The genes currently approved as *VPS13A* (vacuolar protein sorting 13 homolog A) and *ATG2A* (autophagy related 2A) are the most well characterised and therefore “founding members” of this newly defined BLTP superfamily. Crystal and cryo-EM structures of fragments of VPS13A and ATG2A [21–24] and full-length ATG2A [25] showed that these proteins fold to form a long rod-like structure with an internal hydrophobic groove ideal for transport of lipids; importantly, purified VPS13A and ATG2A are both capable of transporting lipids between liposomes in vitro [21, 22, 26–28].

The predicted structures of VPS13A and ATG2A from AlphaFold [13] showed remarkable similarity to the experimental structures of these proteins [29]. Further analysis of these predicted structures led to the identification of a simple modular unit that contains five antiparallel β-strands curved to form a “U-shape” followed by an unstructured loop that curves back across the β-sheet. This modular unit is called the repeating β-groove (RBG) domain [29]. The inner concave surface of each RBG domain is lined primarily with hydrophobic residues, while the outer convex surface is primarily hydrophilic. Head-to-tail multimerisation of RBG domains leads to the formation of proteins with a long hydrophobic groove (Fig. 1). In cultured cells, VPS13A and ATG2A localise to membrane contact sites [21, 25, 26], where they form bridges that connect two organelle membranes and allow non-vesicular lipid transfer [29, 30], leading to our decision to name this gene family the ‘bridge-like lipid transfer protein’ (BLTP) family.

Notably, the KIAA1109 protein is also composed of RBG domains, and thus is another member of the BLTP protein superfamily [29]. The defects in subcellular PI(4,5)P_2_ distribution in fly *tweek* mutant cells [5] are also consistent with a lipid transfer function for this protein.

We briefly considered assigning RBG as a root symbol to this newly identified gene group, in reference to the repeating β-groove domains in their encoded proteins, but this clashed with the nomenclature for two unrelated yeast genes, *RBG1* (RiBosome interacting Gtpase 1) and *RBG2* (RiBosome interacting Gtpase 2) and would also have been less functionally informative than the BLTP nomenclature. Thus, *KIAA1109* has been named *BLTP1* (bridge-like lipid transfer protein family member 1).

## Additional members of the BLTP superfamily

Alongside *KIAA1109*, the BLTP family also includes another gene approved with an uninformative placeholder symbol: *KIAA0100*. This gene has not been extensively published on to date, although its orthologs in yeast and fly have been better studied. The fly ortholog of *KIAA0100* is named *hobbit*, since mutant animals have a substantial reduction in body size [31, 32]. Fly *hobbit* mutant animals exhibit cell-autonomous defects in regulated exocytosis, and the small body size of the mutant animals is caused by failure to secrete insulin [31]. In flies, insulin is a potent regulator of growth, analogous to *IGF1* in vertebrates [33]. There are two paralogs of *hobbit* in *S. cerevisiae*: *FMP27* (alias *HOB1*) and *HOB2* which are both orthologs of *Drosophila HOBbit*. The fly and yeast “Hobbit” proteins both localise to endoplasmic reticulum-plasma membrane (ER-PM) contact sites [15, 32], and the subcellular distribution of PI(4,5)P_2_ is disrupted in fly *hobbit* mutant cells [32]. Naming this gene in vertebrates after the fly “*hobbit*” could be viewed as pejorative and hence was not an option. Thus, *KIAA0100* has been named *BLTP2* (bridge-like lipid transfer protein family member 2)*.*

The final two members of the BLTP family were approved as *UHRF1BP1* (UHRF1 binding protein 1) and *UHRF1BP1L* (UHRF1 binding protein 1 like) in vertebrates. However, the alias symbol “SHIP164,” standing for Syntaxin 6 Habc Interacting Protein of 164 KDa [34, 35], has also been used several times to refer to the protein encoded by *UHRF1BP1L*. This protein has been shown to localise to endocytic compartments, and purified protein is capable of transferring lipids between liposomes in vitro [35]. We propose to update the nomenclature of these two genes to utilise the BLTP root symbol and have named them as *BLTP3A* and *BLTP3B*, respectively.

Although *ATG2A*, *ATG2B*, *VPS13A*, *VPS13B*, *VPS13C* and *VPS13D* are all members of the BLTP gene group, their approved nomenclature has not been updated. This is because the existing nomenclature is already functionally informative, well published in the literature and in line with the yeast orthologs of these genes. In addition, variants in *VPS13A*, *VPS13B VPS13C* and *VPS13D* have all been associated with human phenotypes (chorea-acanthocytosis (MIM: 200150), Cohen syndrome (MIM: 216550), early onset Parkinson disease (MIM: 616840) and spinocerebellar ataxia (MIM: 607317), respectively). The HGNC now strives to stabilise the nomenclature of symbols associated with disease phenotypes where possible, especially when they have already been used in clinical publications. However, we were able to assign these genes BLTP# alias symbols (see Table 1).

**Table 1:** A summary of the HGNC approved nomenclature of genes in the BLTP superfamily in human

HGNC id	Approved Symbol	Previous Symbol(s)	Aliases
26953	***BLTP1***	KIAA1109	FLJ21404, FSA, KIAA1371, Tweek
28960	***BLTP2***	KIAA0100	DKFZp686M0843, MGC111488, BCOX1, CT101, BCOX, FMP27, Hob
21216	***BLTP3A***	C6orf107, UHRF1BP1	FLJ20302, dJ349A12.1
29102	***BLTP3B***	UHRF1BP1L	KIAA0701, SHIP164
20928	*ATG2A*		KIAA0404, **BLTP4A**
20187	*ATG2B*	C14orf103	FLJ10242, **BLTP4B**
1908	*VPS13A*	CHAC	KIAA0986, **BLTP5A**
2183	*VPS13B*	CHS1, COH1	**BLTP5B**
23594	*VPS13C*		FLJ20136, FLJ10381, KIAA1421, **BLTP5C**
23595	*VPS13D*		FLJ10619, KIAA0453, **BLTP5D**

BLTP-approved symbols and aliases are highlighted in bold

It is a fairly common issue in nomenclature that one or more very highly published symbols for members of a larger gene group need to be retained, while there is still a great benefit to assigning the rest of the set new, functionally informative nomenclature. In these cases, the genes getting new approved symbols are given the first numbers in the series and the genes that are retaining their current nomenclature but are being assigned group aliases come later in the series; this convention was followed for the BLTP family members. If the genes changing to a new root symbol were given the later numbers, for example if *BLTP3* was an approved gene symbol but *BLTP1* and *BLTP2* were assigned as aliases, it could be confusing, perhaps leading researchers to mistakenly assume that the first genes in the series might be named in other species with no human orthologs.

We wrote to all authors who had previously published on *KIAA1109*, *KIAA0100*, *UHRF1BP1* and *UHRF1BP1L* about the proposed BLTP update to gauge their support for this new nomenclature. The majority of respondents to our nomenclature proposal consultation were in favour of this update and so the changes were made as detailed in Table 1. We also proposed assigning BLTP aliases to *ATG2A*, *ATG2B*, *VPS13A*, *VPS13B*, *VPS13C* and *VPS13D*.

An HGNC gene group for the ten human genes encoding “bridge like lipid transfer proteins” can be seen on our website (https://www.genenames.org/data/genegroup/#!/group/2141).

This new BLTP nomenclature is functionally informative, and we hope that as researchers in the field continue to characterise this set of lipid transporters that they will adopt it in their publications.
